# Exploring the consent process among pregnant and breastfeeding women taking part in a maternal vaccine clinical trial in Kampala, Uganda: a qualitative study

**DOI:** 10.1186/s12910-024-01055-7

**Published:** 2024-05-16

**Authors:** Agnes Ssali, Rita Namugumya, Phiona Nalubega, Mary Kyohere, Janet Seeley, Kirsty Le Doare

**Affiliations:** 1 Medical Research Council/Uganda Virus Research Institute & London School of Hygiene and Tropical Medicine Uganda Research Unit, Entebbe, Uganda; 2 Makerere University -John Hopkins University Research Collaboration, Kampala, Uganda; 3 St George’s, University of London, London, UK; 4 London School of Hygiene and Tropical Medicine, London, UK

**Keywords:** Consent, Pregnant women, Maternal, Vaccine, Clinical trials

## Abstract

**Background:**

The involvement of pregnant women in vaccine clinical trials presents unique challenges for the informed consent process. We explored the expectations and experiences of the pregnant women, spouses/partners, health workers and stakeholders of the consent process during a Group B Streptococcus maternal vaccine trial.

**Methods:**

We interviewed 56 participants including pregnant women taking part in the trial, women not in the trial, health workers handling the trial procedures, spouses, and community stakeholders. We conducted 13 in-depth interviews and focus group discussions with 23 women in the trial, in-depth interviews with 5 spouses, and 5 women not in the trial, key informant interviews with 5 health workers and 5 other stakeholders were undertaken.

**Results:**

Decision-making by a pregnant woman to join a trial was done in consultation with spouse, parents, siblings, or trusted health workers. Written study information was appreciated by all but they suggested the use of audio and visual presentation to enhance understanding. Women stressed the need to ensure that their male partners received study information before their pregnant partners joined a clinical trial. Confidentiality in research was emphasised differently by individual participants; while some emphasised it for self, others were keen to protect their family members from being exposed, for allowing them to be involved in research. However, others wanted their community participation to be acknowledged.

**Conclusion:**

We found that pregnant women make decisions to join a clinical trial after consulting with close family. Our findings suggest the need for an information strategy which informs not only the pregnant woman, but also her family about the research she is invited to engage in.

**Supplementary Information:**

The online version contains supplementary material available at 10.1186/s12910-024-01055-7.

## Background

Groups are considered vulnerable in the research setting when they have a compromised ability to protect their interests and provide informed consent. Pregnant women may have agency to protect their own interests and give their own informed consent in clinical vaccine trials [1], but they are also responsible for protecting the interests of the growing foetus, who cannot consent to research or may have a unique susceptibility to risks. There are additional distinctive issues that a pregnant woman, and her family, may consider regarding the risks and benefits of participation in clinical research, resulting from the interdependence between mother and foetus. In a patriarchal system, she carries a child who is the continuation of the spouse’s blood line [2]. So, decisions about the foetus are not hers alone to make. Even though the interests of the mother and the foetus are conceptually separable, in practice, they are considered together.

Pregnancy and infancy are both periods of increased vulnerability to infection [3]. Vaccinating women during pregnancy has been shown to be effective in providing protection against several infections in pregnant women, while also providing protection for the foetus and the infant during early life [4]. Although a small number of vaccines are recommended for routine use during pregnancy, there are many vaccines that have sufficient safety data to support their use in pregnant women in appropriate circumstances [5]. Despite the benefits, a lack of vaccine confidence remains a significant barrier to vaccine uptake among pregnant women worldwide. This has been a particular challenge during the COVID-19 pandemic, which has seen low rates of vaccine uptake among this cohort [6–10] .

We have recently shown that in Uganda, vaccine uptake in pregnant women was influenced by the awareness of the vaccine, disease severity and susceptibility, vaccine benefits, side effects and risk of harm during pregnancy, history of previous vaccination, and recommendation from healthcare professionals [11]. We found that there was a need to include the wider community in vaccine discussions to increase confidence in existing vaccines. Communicating such information clearly is even more vital during trials of investigational vaccines. Given the benefits of the protection afforded by vaccination for pregnant women there is a growing awareness that pregnant women should be included early in clinical trials. How information on the trial is best provided is critical to vaccine confidence both for the woman herself, her family and for the wider community.

An important part of the sharing of information is the informed consent process. Many factors can influence a research participant’s understanding and experience of the information provided, such as the type of study, the cultural setting, local beliefs and customs, as well as the participant’s language, religion, level of education, and socio-economic status [12]. In a maternal vaccine trial, women in different socioeconomic situations and with different health-care experiences may have very different information needs about the disease that the vaccine is protecting against and the safety and efficacy of the vaccine [13].

As noted above, a pregnant woman is not necessarily the sole decision-maker for matters affecting the foetus she carries. While care should be taken to ensure that decision-making about participation in a trial does not undermine the autonomy of pregnant women, not planning for third-party consent could be a major encumbrance for research. In this study we explored the expectations and experiences of pregnant women of the consent process in Uganda. We also explored the attitudes and experiences of health workers and partners/spouses of the women and other community stakeholders. Our aim was to learn what information is required during the consenting process and in what format, to allow pregnant women and their family, to make informed decisions before joining a clinical trial.

## Methodology

### Study design

This cross sectional exploratory qualitative study was embedded in an ongoing maternal vaccine clinical trial (CTA 0212) conducted by Makerere John Hopkins University Uganda limited (MUJHU) in Kampala, Uganda.

The clinical trial in which the study was conducted was a phase II study of a multivalent vaccine against the Group B streptococcus (GBS) capular polysaccharide (CPS) in pregnant HIV-infected and uninfected women. For the trial women were enrolled from two main health facilities, Kawempe national referral hospital and Kisenyi health centre IV located in the Centre of Kampala city (the study details are available at https://clinicaltrials.gov/study/NCT04653948?locStr=Uganda&country=Uganda&term=maternal%20vaccine&rank=2).

The study was conducted at Kawempe specialised national referral hospital for gynaecology and obstetrics. It is located within 4 km from the city centre in one of the administrative divisions of Kampala city. The division has several neighbouring communities including Mulago, Kamwokya, Komamboga, as well as Kawempe where most of the stakeholders interviewed lived. The hospital receives women from all over the country who may be referred, as well as serving nearby communities.

### Theoretical background to the sampling

The theoretical underpinning used in this study is the socio ecological model of health [14, 15]. The model portrays the relational influences on an individual showing how the family and wider society may impact on an individual’s actions and decisions [16]. In this study we view the woman as being influenced by her close family relations, the family belongs to a community which has cultural beliefs and social norms. In this study the women could legally make their decisions from the age of 18 years; however, because they were pregnant, the decision to enrol in a maternal vaccine trial was influenced by others, notably their family.

### Recruitment

Pregnant women were recruited from the hospital after administrative permission and individual consent had been obtained. Pregnant women who were taking part in the Group B Streptococcus vaccine trials were approached following contact from the clinic research team. Interested participants were contacted by the social scientists by phone and were informed about the study and requested to come to the hospital for detailed information during their follow up visits at the antenatal clinic. The social science researcher identified pregnant women already enrolled in the trial who were returning for their second follow up visit at the clinic. Some were pregnant while for others it was a post-natal visit. These visits did not include extensive clinical procedures and therefore allowed time for the interviews or discussion.

The inclusion criteria for this qualitative study were any pregnant woman aged 18–39 years at any gestation who was taking part in a maternal vaccine trial conducted by MU-JHU and willing to give consent to take part. The study also included breast feeding women in the same age bracket who was taking part in follow up of the trial. The social science team purposively sampled the pregnant women and breast-feeding women in different age brackets (18–24 years, 25–32 years, 33–39 years) from the lists provided by the data management team.

An additional group of women attending the same antenatal clinic at the referral hospital who were not in the clinical trial but had similar characteristics as those outlined in the clinical trial inclusion criteria, and willing to give consent were invited to participate in an interview. These women were purposely selected from those attending the antenatal visit to match the age of women in the trial 18–39 years. They were recruited through the antenatal clinic staff. A midwife introduced the qualitative study’s main objective and requested the women interested in discussing the study in detail to meet with the social scientists. The social scientists shared the details of the qualitative study and women who were willing to join and sign a consent form were invited to take part. The women were interviewed in a private area in the hospital space.

Partners to women were recruited with the support of their wives. The social scientists obtained the partners’ contacts from the wives and contacted them by phone and invited them to take part in the study after giving written informed consent. The partners were free to suggest their preferred location for the interview.

Community stakeholders were mobilized with the support of a Community Advisory Board (CAB) member. The CAB member provided the research team with contact information for the stakeholders, who were drawn from community leadership structures, and included political, cultural, religious, and civic leaders. They were contacted by phone by the social scientists and later visited for a discussion of the participant information document. The stakeholders were interviewed at the time and place of their choosing after giving consent. The research ethics committee member was purposely contacted and invited to take part in the study.

The social science team purposely approached healthcare workers who were involved in the trial processes and requested their participation in the study. The health care workers were selected because they obtained consent from the women, made home visits to the participants, did the vaccination procedure, and offered treatment and care for the participants.

### Data collection

Data collection took place from October 2022 to Feb 2023. The women in the trial either took part in a semi-structured Individual Interview (IDI) or a Focus Group Discussion (FGD). In-depth interviews were conducted to explore individual real-life experiences of the women. FGD were conducted among the women to explore the general community experiences of the informed consent process for maternal vaccines. The women who took part in the in-depth interviews did not take part in the FGDs. All the women were aged between 18 and 49 years. Insights from the stakeholders who included spouses, community leaders and health workers were gathered through in-depth interviews. By asking the different groups of respondents the same research questions we were able to explore the research questions from different perspectives [17].

Verbal permission to audio record the interviews was requested before conducting the interview. The interview guide for the IDI and FGD topic guide (see Supplementary File 1) included topics that included antenatal visit experiences, knowledge of vaccines in general and the specific vaccine they received, barriers and facilitators for taking part in the clinical trial, what the consent process involved, study information shared with them and the format of presentation, and decision making to join a clinical vaccine trial. The individual IDI lasted between 30 and 60 min. The FGDs lasted between 60 and 90 min. The interviews were conducted by two female social scientists. The FGDs were conducted by three female social scientists with one as an observer. The individual interviews and FGDs were conducted in English and Luganda languages depending on the preferred language of the individual and group. The interviews with women in and not in the trial were conducted in a private space at the hospital. The FGDs were conducted within the hospital in large office spaces. During the FGDs, participants were served a soft drink and a snack.

Stakeholders chose the place where the interview was held, some came to the hospital premises, while others were interviewed in their communities. The health workers were interviewed at the study offices at Mulago hospital and Kawempe referral hospital. The interviews with the stakeholders and health workers lasted between 30 and 50 min The first author listened to a sample of audio-recordings throughout the study to follow up and discuss probes and emerging findings during weekly debriefing sessions.

### Data management and analysis

Once a participant was interviewed and after an FGD had been conducted, the recording was uploaded on an encrypted computer. The two social scientists who collected the data did the transcribing and translation. Luganda transcripts were translated and transcribed by the same social scientist into English because the research team members were all conversant with both languages and had skill to do this. The two social scientists transcribed each other’s interview so that they transcribed an interview they did not conduct. This was helpful in generating questions to ask each other about the findings, check reliability and improve interviewing skills throughout the duration of data collection. Listening to each other’s recording and the lead researcher listening to the recordings led to the writing of analytical memos on the themes from the data, which were useful during analysis.

All the transcripts were anonymized. Identification numbers were assigned to every transcript, and these were securely saved on the MRC server in Entebbe.

Thematic data analysis was employed for this study [18]. The team started with familiarizing themselves with the data by going through several transcripts each, and actively looking for patterns from the data which led to codes and they compiled a code book which was then used to code all the data sets. During the analysis, the team began by discussing the first five transcripts to come up with codes for the data set, this was a continuous process after the first five transcripts until the team members agreed that no unique codes were emerging. The discussion of codes followed the questions in the interview guides, the research team agreed to code freely to allow for topics which emerged during the interview.

After developing the codes and the codebook, data were exported to NVivo 12 an electronic qualitative data analysis software to support the analysis process. Data analysis was both deductive following the questions in the topic guides and inductive from within the data. The themes identified include: perceptions about vaccines in general, perceptions of the vaccine used in the overarching clinical trial, experiences at the antenatal clinic, decision making, barriers and facilitators to enroll in a maternal clinical trial and discussion of the elements of the consent process (study information and presentation, role of an impartial witness, confidentiality, compensation, and strategies for information sharing).

### Ethics approval and consent

After mobilizing the study participants, the research team provided detailed information in English or Luganda, the local language, depending on the language preferred by the participant. Participants were given an opportunity to ask questions about the planned study. All the participants gave their individual informed consent by giving written consent. If a participant was not able to read and write, the researchers involved a peer attending at the clinic on the same day, or male partner if he had escorted his wife to the clinic and could read and write. The partner or peer were part of the information sharing session and the peer or partner signed as a witness after the volunteering participant had given a thumb print.

The quotations are assigned identifiers, FGD/IDI for mode of data collection. For each FGD we then provide, its number, and age of participants. WIT is an abbreviation for `Women In Trial’, WNT is `Women Not in the Trial’. IDIs are shown by the participant identifier and age.

## Results

Fifty-six respondents took part in this study, these included thirty-six pregnant or breastfeeding women, 5 women not in the trial, 5 partners to women in the trial, 5 health workers (1 male, 4 female) and 5 community stakeholders (2 male, 3 female). The pregnant and breast-feeding women were aged 18–39 years although most were aged below 25 years. Male partners were older, aged 30–49 years. The stakeholders were aged between 30 and 50 years.

All the participants had some school education. Occupations of the women included farming, small businesses such as hair salons, and some women said that they were `housewives’. The stakeholders were leaders in their communities, one of whom was a practicing health worker. The demographic information of those who took part is shown in Table 1.

Health workers were aged 28–33 years, they had experience of conducting maternal clinical trials for a duration of between 2 and 4 years. The health workers had attained diploma and degree level qualifications.

**Table 1:** The demographic characteristics of the respondents in the study without the health workers

Characteristics	Pregnant women enrolled in trial- Interview. (*N* = 13)	Pregnant Women enrolled in trial-FGD. (*N* = 23)	Pregnant Women Not in trial (*N* = 5)	Male Partners. (*N* = 5)	Stakeholders. (*N* = 5)
**Age**					
17–24	8	8	0	0	0
25–29	3	6	3	0	0
30–39	2	9	2	4	1
40–49				1	
≥ 50					4
**Sex**					
Male	-	-	-	5	2
Female	13	23	5		3
**Gestation**					
6-7months	2		2		
7-8months	8		0		
8-9months	1		2		
≥ 9months.	1		1		
Marital status					
Married	11	23	5	5	
Single	2	0	0	0	
**Level of education**					
Primary	1	-	2	1	
Secondary	8	-	2	1	2
Diploma	1	-	0	0	0
Tertiary/ Certificate.	2	-	0	3	1
Bachelors’ Degree.	1	-	1	0	2
**Occupation**					
Religious leader.	-	-	-	-	1
Village Health Team					1
Local council Leader.	-	-	-	-	2
Business/Trader	6		1	2	
Peasant Farmer	1			1	
Saloon	2				
Tailor					
Health Professional	-	-	-	-	1
Housewife	2		3		
Teacher/librarian	1				
Electrician	-	-	-	1	
Office Attendant	-	-	-	1	
Unemployed	1		1		

### The consenting process

For most pregnant women the decision to join the trial involved the husband as a prerequisite to joining because she was carrying the pregnancy of the male partner. All the women reported how they were informed about the research and said that they were usually given a week to discuss the study document with their family members. A woman taking part in a focus group discussion:

*The time they gave me the study information, my husband was working in a far place so I could not consent on my own. They gave me information sheets that I went with at with home. When my husband came back, I showed him the sheets and later he allowed me to participate.* (FGD-002- [30–35 years]-WIT).

Another woman in another discussion said she had made the decision herself, after receiving additional information:

*For me, like I told you that the first time I came I did not understand, I came back again, and they explained then I understood and decided to join, for me, l asked my heart.* (FGD-003- [25–29 years] -WIT).

During an interview a woman, who was not in the trial, explained the rationale for seeking her partner’s consent to take part:

*I would follow his opinion because I might be wrong, if we both agree there would not be any problems, but if he disagreed, I would follow what he[husband] says. Because I’m his responsibility, that’s why if I talk to him about it and he says no, I can’t do otherwise, his opinion is very important in my decision making.* (IDI-004-WNT).

The health workers’ responses were similar to what the women had shared, the health workers emphasised the importance of sharing key study information and letting the women share study information with their networks such as partners/husbands.

Other stakeholders observed that informing partners was important. A village health team member commented that they thought that: ‘*Most pregnant women don’t disclose to their husbands that they are participating in clinical trials. When their partner finds out that they are participating in clinical trials, it may lead to domestic violence*.*’*

Most pregnant women consented to take part in the trial for the benefit of themselves and their unborn child. The women had the belief that they would give birth to healthy babies without infections, an example, *‘Because I saw that, the way they explained the vaccination just like I told you my baby and I were going to be safe’.* (IDI- -002-WIT).

When asked about the consent procedure many respondents mentioned risks and benefits of the study, purpose of the study, side effects of the vaccine and what may happen if a participant wanted to withdraw from the research, as being very important pieces of information that every participant needed to know.

A male participant mentioned the need to present information about safety:

*I would like to know the information on the safety of that research. I would prefer hearing information like “a certain number of pregnant women participated in this research and both mother and baby are all safe.” That kind of information must be approved by the Ministry of Health.* (IDI- -001-MALE PARTNER).

There was a consensus among those interviewed, and in the discussion groups, that the information shared needed to be clear, and free from technical language. In addition, the information needed to be given by someone who understood the trial and who could answer any questions.

All the respondents stressed that participants in research must sign the consent form because it was to prove they understood the information and willingly consented to take part: ‘*It shows proof that you consented to take part in the study without being forced. Signing means that you agreed to take part without being forced’*. (FGD-001-[18–24 years]-WIT).

The women in the trial commented that providing consent is not uncommon in most transactions. One woman compared the process to the savings associations in their communities where a woman agrees to the terms of that group, when she becomes a member. She went on to explain why signing the consent form is important in research:

*They [research participants] might get a challenge from elsewhere and they accuse the researchers saying “they even forced me to join the study” but if they completed that consent form and signed it very well, the researchers have proof and evidence on their side that that person agreed to take part and even signed the document, that is why we sign on those documents.* (FGD-001-[18-24years]-WIT).

Several women who by now had babies reported that they had referred back to the information documents during the trial especially for procedures that had to be carried out on their babies. During the FGD with older women one woman noted that she had kept the study information document with her every time she visited the clinic:

*For me I used to come with it in my bag every time I had a visit, I used to take my time reading it while waiting to see the doctors, it helped me follow through very well, I could tell that this is what they are doing at this stage, just to prove that what they are doing is what they taught me at the beginning*. (FGD-002- [30-35years]-WIT).

Another mother referred to the document because she was being told to express breast milk:

*For me when they told me to extract breast milk the very first time in the study, I asked the doctor why they were taking my breast milk, the doctor told me that all that information was explained to me during the information session before I joined the study… in brief that forced me to go back and look critically at the copy they gave me here, that’s how I understood that its true they had to collect breast milk on the first day I gave birth.* (FGD-002- [30–35 years]-WIT).

A blood draw from the baby led another mother to refer to the study document:

*I read it several times when they had just given it to me, then I kept it somewhere safe, I had seen everything even collection of breast milk on the first day, but what I didn’t see, was collecting blood from my baby, after two weeks they tell us to bring the baby and they take off a blood sample from their little hand, that really hurt me and I thought to myself ‘why do they take off all that blood from such a little baby’, I was puzzled; I went back and picked the paper and read it carefully to see whether it was in that paper, I found the information there and understood why the doctors did it.* (FGD-002-[30–35 years]-WIT).

It was clear that many trial participants understood the significance of signing the consent form, as well as the importance of retaining the information sheet for future reference.

### Comprehension of study information

The respondents were asked about the best practice for sharing study information. They mentioned several tools or ways that would enhance comprehension of clinical trial information. These included the use of short video clips, picture/poster presentation, recorded audio study information, study flyers and the use of trial participants providing study information with each other. They also suggested the use of media which may include radio and television and social media communication like Facebook and WhatsApp which could enhance understanding for both the literate and less illiterate participants.

The following FGD excerpt shows some suggestions from women aged 18–24 years:

P1: *… If you also share comparisons of the women who get vaccinated and those who don’t get vaccinated, it can make the woman make an informed decision*.

P5: *I think broad casting that information on a TV is beneficial. When a woman reaches the hospital, she can be watching that information on the TV before the health workers attend to her.*

P8: *Most information now is passed on phone and TV. I think you can get a program on any media platform and share such information. Even when you come at the hospital, you can put up a billboard where you can put such information.*

In an older age discussion group one woman commented:

*Remember there are some of us who have good experience about this [vaccination], I think it can be very good if we share with other women and help them decide. You can also do that in the different villages, on different radio programmes, remember people listen to these radios, they can find this information there, and create awareness in the communities.* (FGD-[30–35 years]-WIT).

Use of posters/photographs was mentioned by some women: `…*yes, the photos can inform a person and the health care worker will explain to you where you don’t understand’* (IDI-002-WIT). A health worker also mentioned the value of visual methods:

*You can use posters, use visual aids with the pictures of what you are trying to explain to them, because this person is not able to read, but they can see that the pictures are directing them to do this and that, so that they can understand, with arrows and different directions, I don’t know how to clearly explain but what I know is that this can really help.* (KII-001- HEALTH WORKER).

Pictures on the information sheet were also suggested by a health worker:

*We could draw some pictures on a sheet of paper may be as part of the information sheet (ICF) at the end, we include the benefits of participation in the study, we draw pictures of the baby being protected against the effects of the Group B Streptococcus infection, I think participants may be able to see the pictures and understand the information compared to the words they can’t read.* (KII-004-HEALTH WORKER).

The same health worker mentioned the use of short video clips to enhance comprehension:

*For the mother who can’t read and write we can put up short videos, because even if a mother can’t read and write but there is a short video that she is able to watch, I think it’s better for them instead of giving them an ICF which they can’t read, if it’s a video they can watch and understand the information being shared.* (KII-004-HEALTH WORKER).

Sharing recorded study information using phones since many women now own phones, was also suggested.

### Role of an impartial witness

We asked respondents to share their perspectives on the role of an impartial witness during the consenting process. The impartial witness, according to our study findings, was viewed by most respondents as a legal requirement to protect the person being researched and the researcher.

One woman during a FGD said that presence of an impartial witness benefited the researcher:

*I think the witness is important in cases where that person who signed or put their fingerprint in agreement to participate in the study initially, later changes their mind about anything against the initial, they can ask the witness because they were around when that person agreed to participate in the study, and they can give evidence that that person was not forced to sign on that document.* (FGD-002-[30–35 years]-WIT).

However, some participants did not think it was necessary to have a witness. They said that a witness may not always be reliable. To these women, understanding information is not limited to reading and writing by the volunteer. An example that was compared to bank transactions emphasizes the need for autonomy once a volunteer understands the language that is being used:

*Many times, we go to the banks to sign documents with people who cannot write but there is an option of putting a thumb print. I think a witness can even reach a point and deny that she signed on your behalf. According to me, a witness is not important.* (FGD-003-[25–29 years]-WIT).

The women in that discussion group went on to note that the independence and privacy of someone who does not read but understands what is read should be respected. ‘*Some things need to be hidden. I may have my own secrets that don’t require me to be with a witness during the consenting process’.*

Finding the impartial witness is not always easy in some contexts and that would mean that an interested volunteer who understood the information shared would not enrol in the research:

*…, getting a suitable witness is not easy, sometimes we use hospital staff, remember they also have things to do yet going to pull her out of their work for one hour, just to sit and listen to you. I think what we can do for some of those cases is we can give them some time to go home and look through that information, and if they have someone at home who is literate, they can even ask them for help, then when they come back it would be a quicker process for them.* (KII-005 HEALTH WORKER).

The stakeholders and male partners had similar descriptions of a witness to those shared by most women, a witness was to prove that a study participant agreed to take part in a research study.

### Compensation of study participants

Compensation for time is another important aspect of the informed consent process. Whereas most participants reported that it was important to compensate research participants, how research participants were to be compensated varied. The women reported they needed the money to help them in travel and they would use some of it to cater for some of their needs back home. For some women it was a reason to stay in the study.

The male partners felt it was important to compensate the women that participate in this research and mentioned it is an additional incentive to encourage retention. There was also the suggestion to give goods as part of the compensation package: *’Apart from giving them cash, you can buy some items for the baby or some home necessities like you do some shopping for the family like food that is necessary for the woman after giving birth’*. (IDI-004-MALE PARTNER).

Other suggestions from the male partners included the need to consider the distance between the study site and the participant’s home before determining transport reimbursement costs, consider that the transport costs fluctuate and therefore researchers should arrange to increase reimbursement accordingly. Men mentioned that when women stay for a long time at the clinic during the clinic visits, they need to be provided with a meal or something to eat.

The health workers reported that compensating for transport costs and time were important to encourage women to attend the frequent visits to the clinic.

### Confidentiality

Women were asked what they understood about confidentiality in research. Some participants in an FGD defined confidentiality in terms of secrecy so that community members never got to know about their involvement in research.

*I think confidentiality means, keeping my secret about participating so that am not despised for people to say that am like a rat they use to test drugs, because that’s how people perceive it, yet they don’t know that it’s to benefit every pregnant woman.* (FGD-002- [30–35 year] - WIT).

For others, it was keeping a secret and not sharing information beyond the two people - researcher and volunteer. Others said that you could share the information without identifying the person:

*The identification numbers they give to us are the ones that help us to remain confidential, that the information we give, even if it goes public, it not easy to know that this and such a person is the one who gave out the information.* (IDI- 003-WIT).

A male partner said he did not care what they shared if they did not include sensitive information about family:

*I don’t think confidentiality is necessary, I don’t think there is any information I want to hide, am so free even if my name is mentioned in public, especially if I know that I don’t have any crime, the only thing I mind about is protecting my family, so they can put my name anywhere, but they shouldn’t put other details like my wife’s name and children.* (IDI-004-MALE PARTNER).

One of the stakeholders mentioned the importance of volunteers’ contributions to the research being included:

*I agree that all the information shared by the participants should be kept confidential, what I mean is no one should be able to identify that these particular views came from the woman leader or attaching our names on views because, this means that we shall be identified, however you can just say that these views are from the leaders of Kawempe in general so that we don’t miss out on sharing our views with you.* (IDI-004-STAKEHOLDER).

### Involving family members in decision-making

Most women said that they accepted to join the vaccine trial after consulting their spouses before a final decision to join:

*When I accepted, I went back and explained to my husband about the research, and he also advised me that it was a good thing being part because he had a friend whose wife has participated in a research study. When I agreed to join the study, I took the consent form for him to read and after he read through, he told me to continue in the study because it was good.* (FGD-001-[18–24 years]-WIT).

Women reported accountability to spouses, one woman took pictures of the information document and sent them through social media (WhatsApp) for him to read before she gave consent.

Some women informed their spouse but also went on to inform their close relatives like their mother before they decided to join the vaccine trial.

*The truth is I did not accept to join that very day, they taught and explained everything, I understood very well but I told the health worker that I will come back, I had to ask my husband, … I went and also told my mother about it and she said most of the medicines they make first go through such trials, even the current vaccines were tested so she gave me a go ahead* (FGD-002-[30-35years]-WIT).

The fear of consequences in the marriage if a woman did not inform the spouse was highlighted by one of the women:

*‘I think its important [to engage a partner] because I don’t want to create misunderstandings within the family, I want a peaceful marriage, because If anything happens to the baby, I am answerable’.* (IDI-012-WIT).

One woman who was not in the trial mentioned the challenge to autonomy caused by the cultural context if a woman wanted to take part in a trial, because a husband might not grant his permission for something a woman may be very keen to do.

Our findings show that some men would seek advice elsewhere before they made a final decision about their family’s health. One spouse said,

*“I told my mother, and she was not happy with my wife accepting to participate, my friend is the one who encouraged me because she told me, she was also once involved in research at Mulago when she was pregnant for her twins and the children are very healthy up-to today they are now seven years. They had to first convince me, at first l had refused.”* (IDI-003-MALE PARTNER).

A stakeholder mentioned the importance of culture, but they also mentioned economic dependence of some women on men and therefore advised that men should be informed about research in which their wives will participate.

*In our country setting men are the heads of the families and they are responsible for taking care of the homes, women on the other hand are home makers and most of them don’t work, they depend on their husbands for all their financial needs and men take care of them, therefore it is hard for these women to do anything without involving their husbands, it’s very important to involve these men, when doing research.* (IDI-005-STAKEHOLDER).

Our findings indicate that while many women felt able to decide on participation in the trial, including seeking more information and clarifications, they were also conscious that they needed agreement from their partners. The men we consulted valued the opportunity to be given information on a trial their pregnant partner may take part in and be part of the decision-making.

## Discussion

The findings of our study show the importance of giving enough time to potential volunteers to reflect and contact their relatives and key stakeholders in their decision-making process. Pregnant women are a special group because they consent for themselves and for their babies. The family and community support systems need to be engaged when sharing research study information, particularly involve male partners because they are usually the heads of the households and source of livelihood for the pregnant women.

Although some women mentioned that they made their own decision to join for the clinical trial, the majority of the women joined the trial after consulting the male partners who were the fathers of the children to be born. Involving men in the information sharing process remains critical for retention during maternal vaccine clinical trials and creating an environment at the hospitals that would encourage their involvement has been suggested in other studies conducted in Uganda [11, 19]. Most cultures in Uganda follow a patrilineal system and most households are headed by men, and this impacts on how health decisions maybe made by some women [20].

The women we spoke to were concerned about the safety of their baby and this is one reason why they valued the detailed information given to them and referred to the information documents given to them at the time of consent. Similar concerns about safety of the baby have been reported in other studies [11, 21].

Our findings showed that less literate volunteers can understand study information if it is presented in their local or language they best comprehend. Similar findings have been reported that volunteers who may not be able to read and write, do understand information conveyed in their language or the language they understand and do not think they need a witness to confirm their consent [12].

Compensation for time and inconvenience to the participant is required but how to compensate is challenging. Providing food and transport costs are seen as beneficial for participation. A study conducted among participants in an HIV vaccine trial reported that some participants reported that they could stay at the clinic longer because of the meals they were receiving [22]. Another study that followed up phase 1 clinical trials revealed that volunteers may think that they can earn a lot from taking part in clinical trials, and take part because of that, which raises the issue of undue inducement [23]. It has also been reported that participants enrolled to join clinical trials for the health and wellbeing benefits as well as providing a way of meeting with peers [24].

In addition, research teams need to ensure study information is shared in a form appropriate for volunteers in each context to enhance comprehension of study procedures especially if the participants may not be literate.

Participants while discussing best practice to enhance comprehension, mentioned video clips, pictures, poster, and media as useful tools. Previous research has made comparisons between the standard consent document, messaging and videos showing that although there was no significant difference in the three models, the volunteers liked the visual methods which helped them retain information better [25]. Besides the digital and manual tools, peer to peer sharing was noted in our study as a possible strategy to increase comprehension and trust in the vaccine studies. Future maternal clinical trials could adopt a similar strategy to involve women who have taken part in concluded maternal vaccine trials to convey information at different phases of a trial to support the consent process. Getting men to discuss vaccine trial information for pregnant women in this context and similar contexts may reduce suspicion about the women’s participation in a trial. The stakeholders included influential leaders in the community, leaders who may influence decisions about research and healthcare workers. In this study we had only one representative from the ethics committee. In future studies it is useful to involve ethics committee members to discuss the ethics of male involvement in the decision-making path of their pregnant partners who join clinical trials.

### Study limitations

The study was conducted in one hospital in the city which is not fully representative of all the pregnant women and stakeholders especially those in the rural communities. However, this is the national referral hospital for pregnant women, and we included women who did not necessarily live in the city but also lived in suburbs and nearby districts to Kampala. We also had very few stakeholders who took part to discuss their insights although we got useful pointers to community engagement.

## Conclusion

We found that pregnant women make decisions to join a clinical trial after consulting with close family. Our findings suggest the need for an information strategy which ensures the continuous sharing of study procedures throughout the course of any clinical trial to increase comprehension. It is important that this information sharing is not only for the pregnant woman, but also her family, particularly the partners of potential participants, so that they are consulted and informed in a way that promotes understanding. Getting stakeholders and policy makers at the national level to discuss challenges that pregnant women face while making decisions to take part in clinical trials would benefit future clinical trials.

### Electronic supplementary material

Below is the link to the electronic supplementary material.

Supplementary Material 1

Supplementary Material 2

## Data Availability

The data that supports the findings of this study are available from the corresponding author’s institution. The data are however, available from the authors upon reasonable request.

## Abbreviations

IDI: In-depth Interview
FGD: Focus Group Discussion
WIT: Women in Trial
WNT: Women Not in Trial
