# Supplementary material for: Exploring the consent process among pregnant and breastfeeding women taking part in a maternal vaccine clinical trial in Kampala, Uganda: a qualitative study
Source: BMC Med Ethics. 2024 May 16;25:57. doi: 10.1186/s12910-024-01055-7 (PMC11097482; doi:10.1186/s12910-024-01055-7)
Supplement: Supplementary file 1 — Supplementary Material 1 [file 12910_2024_1055_MOESM1_ESM.docx]

**Supplementary file 1.** Individual interview/FGD guide

**Topic Guide 2: In-depth interview Topic Guide for pregnant mothers**

1. Date: _______________________________________________________________________
2. Beginning time: _______________________________________________________________
3. Ending time: __________________________________________________________________
4. Name of the Interviewer: ________________________________________________________
5. Interviewee ID:_________________________________________________________________
6. Age __________________________________________________________________________
7. Level of education _______________________________ *(Ask specific class, tertiary education)*
8. Place of residence_______________________________________________________________
9. How old is your pregnancy ________________ *(Ask in weeks or months depending on what is easy for respondent to respond to).*

**Introduction**

*►Welcome participant and introduce yourself, state the reason for the interview (This study is aimed at exploring your personal experiences, thoughts and feelings about maternal immunization and the consenting process during the clinical trials in which you are taking part)*

*►Explain to participant that their taking part in this interview is voluntary and that participants’ names and individual opinions will not bare their names and the written findings from this study will be bear anonymized names.*

*Introduce the voice recorder (show it to participant) and let them consent verbally to agree to its use during the interview. If they do not accept its use, inform them that you will take notes.*

**Topic 1: Introduction of participants:**

Can you please tell me about yourself? You may tell me anything about your children, how you feel with the current pregnancy or anything that is different since you got this pregnancy (this maybe work, social life among other things)

**Topic 2: ANC attendance experience**

1. What in your view should women do to remain healthy when they are pregnant? What have you done to remain healthy since you got pregnant
2. What are the reasons for why pregnant women attend ANC?
3. Can you please describe what happens during an antenatal clinic visit? ***(Probe/ask about procedures, health education by health workers, any other procedures. What has been helpful to you as you attended the ANC clinic)***
4. What do you usually talk about with peers (fellow pregnant women) when you meet here at the clinic?

**Topic 3: Knowledge of vaccination**

1. What are vaccines? What is the reason people are given vaccines? Which vaccines do you know?
2. Which vaccines do you know of that are given during pregnancy?
3. What in your view are the benefits of vaccinating you as a pregnant mother, what is the benefit to the baby if you as a mother you are vaccinated? Do you have any fears of being given a vaccine while pregnant, what are those fears?
4. Have you ever received any information or materials about vaccination in pregnancy? ***If yes, ask from whom and what type of information did they receive?***How was that information useful to you? ***(Ask her to share what she learnt from the information that was shared with her)***

**Topic 4: Knowledge about pregnant mother’s clinical trials**

1. You are one of the pregnant women involved in research here at Kawempe referral hospital What do you know about this research? ***(Ask about main objectives, procedures of the clinical trial, source of information/who gave her information)***
2. What do you expect to benefit from taking part in the clinical trial? What fears or challenges have you experienced since you joined the trial?
3. What information do you think you should have been told about before joining the research? How best should information about the research be presented to you before you agree to join? ***(Probe for forms of presenting information such as written, video, flip charts any other).***

**Topic 5: Decision making to join clinical trials**

1. How did you make the decision to join the trial? ***(Probe for what it took for them to make a decision, how long after receiving information did they join, did they seek opinion from anyone else, who was that and why did they seek for their opinion)***
2. Since the initial decision to join, what other decisions do you have to make, who influences these decisions, and why
3. What has motivated you to continue taking part in the clinical trial?

**Topic 6: The Consenting process**

**We are now going to talk about the process of getting into the trial and remaining in the trial**

1. What procedures did you go through before you joined the clinical trial? ***Probe/Ask about how they received the information- who shared it with them, how did they consent- ask what is involved when consenting, (reading or listening to study information read to them, signing document, asking and answering questions)***
2. In your view what is the role of a witness during the consenting process of mothers who do not write or read?
3. What factors enabled you as a pregnant woman to take part in the clinical vaccine trial? ***(ask about information shared, information source, support network, culture, partner involvement among others)***
4. What difficulties have you faced while taking part in the clinical trial? ***(Probe about information received, health worker’s communication, peer pressure, social network, spouse and close family involvement, culture (ask participants to specify examples if any).***
   Do you have any fears? ***(Ask her to explain)***
5. In research the participant is told that “all the information you share will be kept confidential” In you view what does this mean?

**Closing**

We have come to the end of the interview; do you have any question about what we have discussed during the interview? Do you have any comment about the research you are involved in?

**Thank you so much for giving your time.**
