# Supplementary material for: Exploring the consent process among pregnant and breastfeeding women taking part in a maternal vaccine clinical trial in Kampala, Uganda: a qualitative study
Source: BMC Med Ethics. 2024 May 16;25:57. doi: 10.1186/s12910-024-01055-7 (PMC11097482; doi:10.1186/s12910-024-01055-7)
Supplement: Supplementary file 2 — Supplementary Material 2 [file 12910_2024_1055_MOESM2_ESM.docx]

**Supplementary file 2. Data summary**

**Data- Theme extracts**

Before trial

They first called us and informed us about the research study. They found us seated and they informed us about that study. After informing us about the study, they later asked us if we had accepted to join. When we replied to them that we had accepted, they later took us under the tent and they informed us about the MINERVAX research, they later brought us information sheets. They used not to force us to join the research, they told us that it was voluntary. They gave us the sheets and provided us with the study information. After giving us the study information, we then decided to participate after realising that the research was beneficial.

They give you a date that is a week ahead for you to decide. On the first day they meet you, they first provide you with the information about the study. Then after providing you with the information, they give you a date which is a week ahead. In that process they give you a phone call to come back with the document and then later they take you for vaccination.

He first gave a health talk about the study and then asked us if we were willing to join and in a different place. I accepted, also another mother, my friend and he gave us the forms that we went through, and he gave us time to go through them even while at home.

Can you briefly share with me how the procedure was for joining the study?

We first introduced each other with the person who interviewed me. She gave me some information sheet that we read together and later asked me to ask her questions where I didn’t understand. I asked her the question and later signed the forms. After signing the forms, she then started asking me questions.

Int: In your view, what do you think would be the most important information to be shared before joining research?

Information about being free to join or not to join research and stopping the participation if I wish to. Secondly, information about privacy. That assures me that my information would be kept confidential.

They told us while we were still at ANC, they picked our files, then called us at side and started telling us everything, they elaborated very well and after it was up to you to either say yes or no but in my group no one said no.

A person is supposed to be told the purpose of research, the benefits of research, side effects and how to deal with the side effects and where to report research issues.

Int: Is there any other information you think would be more useful to share before one joins research?

One may want to withdraw from the research. Therefore, information on what may happen if a person withdraws from research should be indicated, information on how to withdraw from research and contacts for people who can be contacted in case of any emergency in research.

In some situations, the person who seeks consent from a potential volunteer must have clear identification as noted by a woman not in the trial.

You have to be with identifiers like IDs, study information sheets and you must be many. If you are alone, I may refuse to join the study. You must come from an organisation that is known. If you come alone, I may not accept. I may think you want to kill me or sacrifice me. You might inject me and get a problem.

The health worker first explained to me, and I understood what they explained to me, and they gave me a copy of the paper of what they explained to me of which I took a copy at home and shared with someone else and both of us understood, that is when I made a decision.

They gave us a form to sign that very day if one was willing to participate in the study, but also told us to go home and ask our partners for permission, because they were already facing challenges with some participants who don’t tell their husbands about the study and later, it becomes a challenge. But also full consent to the study comes in later after the results are back and everything Is fine, they will call you and ask again have you decided, either yes or no!

The information we usually share is ideally what is in the consent document, so we tell them the purpose of the study, who qualifies and who doesn’t qualify for the study, we tell them the objectives of the study, for how long we are going to follow them up during the study, we tell them the benefits and risks of the study, we also emphasize the need for follow up and why we require them to delivery at Kawempe facility.

So, when we get these pregnant women first of all we pre-screen them, that is we give them a brief introduction about the study, we tell them quickly about what the study is about, and we also tell them about the initial inclusion criteria, if they willing to be contacted by health visitor, willing to deliver from Kawempe hospital or Kisenyi HC, and if they are willing to let their new born infant a participant in the study, if they are willingness to attend all the study visits, if they agree to all that then we proceed to the real consenting process , we go through the ICF word by word and if they have any questions, you attend to them.

I told my mother, and she was not happy with my wife accepting to participate, my friend, but that one encouraged me because she told me, she was also once involved in a research at Mulago when she was pregnant for her twins and the children are very healthy up-to today they are now seven years. And that child is very bright. They had to first convince me, I had first refused.

I wouldn’t feel good about because, I would like you to first tell me that this vaccine we are giving you is for this and that, I want you to tell me why your giving me the vaccine and for what reasons?

I just want to know why they have chosen me to take part in the study.

I think, I have to first do my personal research to find out if it is true, not. After finding out what the truth is, then I also involve my husband and my parent then I can decide.

The participants had HIV and they were going to put them on ARVs. So they were monitoring them minus ARVs. Later they got those sicknesses and she ended up blind. By the time she started ARVs, it was already late. She had become blind. Then when we went on to ask, they were like; I was on such and such a study but she didn’t have a consent form. She had thrown it away. That is why a consent form is very important.

A person is supposed to be told the purpose of research, the benefits of research, side effects and how to deal with the side effects and where to report research issues.

Int: Is there any other information you think would be more useful to share before one joins research?

One may want to withdraw from the research. Therefore, information on what may happen if a person withdraws from research should be indicated, information on how to withdraw from research and contacts for people who can be contacted in case of any emergency in research

DURING CONSENTING

**This period is a kind of mix up with information given and consenting /signing on paper and conduct of procedures at screening before enrollment and vaccination.**

Yes, we read to them because this information is all in the consent document. We usually tell them what is expected and what we need from that person and what is not expected of them.

First they took my blood and urine samples, the doctors wanted to know what conditions did I have before and even after the vaccination they took my samples, so they wanted to that during vaccination what challenges did i have before and after vaccination what challenges did, I have. So, the discovered that I didn't have any complications.

They first test for illnesses such as hepatitis B. they ensure that you don’t have any past history of a medical condition for example having a still birth, pressure or diabetes. They have to ensure that you are healthy before vaccinating you. That is why they draw your blood to first go through screening.

At first they teach you, and then you sign, they did not give us the vaccine immediately, they first tell off your blood sample, they take off blood, on three rounds before they vaccinate you, they have to take off a urine sample to check your health on that, they take off samples from our private parts, after they vaccinated us that very day. After taking off the tests that is when you share

AFTER CONSENTING

First they took my blood and urine samples, the doctors wanted to know what conditions did I have before and even after the vaccination they took my samples, so they wanted to that during vaccination what challenges did i have before and after vaccination what challenges did, I have. So, the discovered that I didn't have any complications.

No, we don’t sign again, they just call you to confirm whether you husband has agreed, and they go ahead. Because they told us, even if you have signed, you’re free to withdraw your consent and you don’t return.

INFORMATION SHARED

We first introduced each other with the person who interviewed me. She gave me some information sheet that we read together and later asked me to ask her questions where I didn’t understand. I asked her the question and later signed the forms. After signing the forms, she then started asking me questions.

Int: In your view, what do you think would be the most important information to be shared before joining research?

Information about being free to join or not to join research and stopping the participation if I wish to. Secondly, information about privacy. That assures me that my information would be kept confidential.

Mainly we use the informed consent form, so as you are going the consent document, you give her a copy to follow through as you read word by word, if there is any part that she has not understood, she is free to ask then you can explain further.

I think, they should be given health talks and teach them about the vaccines, the diseases they vaccinate them against and the effects of not getting vaccinated.

Okay, what is most important of course is the risks and the benefits, you have to explain to them because most of them join thinking that the study is going to solve all their problems so you have to clearly explain the direct and indirect benefits then you also explain the risks and state if they will be minimal or they are high.

**Int:** In your view what is least usefully in the information you share with them?

In a clinical trial everything is needed, we take everything as crucial.

I would wish to know if the research is lawful.

Then it’s always easy for someone to make a decision after knowing the benefits and risks in the study, because if you only tell them the good things, it’s like you have forced them to join the study.

A person is supposed to be told the purpose of research, the benefits of research, side effects and how to deal with the side effects and where to report research issues. One may want to withdraw from the research. Therefore, information on what may happen if a person withdraws from research should be indicated, information on how to withdraw from research and contacts for people who can be contacted in case of any emergency in research.

I don’t think there is any information that should be withheld from the participants, even the risks of participating in the study, however their personal information should not be shared with other people.

It would be better if the health workers themselves come to deliver the information themselves. They can leave the community members to do the mobilisation but if it is giving technical information, then they should be the ones to give the technical information because sometimes they have questions to ask but that person will not answer it adequately. They give wrong answers and the community goes with that.

I think try to use the people they are familiar with, like new you the research is new and this thing is also new, these women can’t trust you, be if the general health worker comes, we have been seeing that person we can easy trust what they have brought for us. For you, you can just come in and add on what they say but let the old health workers share the information about the new research.

I think the person, in charge of that research should be the one to share that information, because I assume they know it better than the other health workers, for example I may come here when am expecting but I have HIV the words that health worker will tell me before I even decide to take the test are the most important whether I know my status or not. They are sometimes words of encouragement that will enable someone make a decision.

**What methods/tools could make sharing of information clearer to volunteers**

You can use posters, use visual aids with the pictures of what you trying to explain to them, because this person is able to read, but they can see that the pictures are directing them to do this and that, so that they can understand, with arrows and different directions, i don’t know how to clearly explain but what I know is that this can really help.

**Int:** Do you use any of those in your current study?

Yes, we have use them especially in the participant’s dairy cards, because after introducing the study drug, we have used the dairy cards to monitor the side effects, and they do this on their own from home, for example we include things like the temperature changes, etc. so for the people who can read and write we have a provision of pictures like the smiling face and a sad face, so they can relate and circle according to how they feel after getting vaccinated with the study drug.

So for the mother who can’t read and write we can put up short videos, because even if a mother can’t read and write but there is a short video that she is able to watch, I think it’s better for them instead of giving them an ICF which they can’t read, they will just look at it and leave it there, but then if it’s a video they can look at it and clearly understand the information being shared.

**Int:** Okay, anything else apart from the short videos?

Apart from the short videos, we can draw some pictures on certain sheet of paper may be as part of the information sheet (ICF) at the end, we put the benefits of participation in the study, we put draw pictures of the baby being protected against the effects of the GBS infection, I think even pictures, they may be able to see the pictures and understand the information compared to the words they can’t read.

IMPORTANCE /MEANING OF SIGNING A CONSENT FORM

It shows proof that you consented to take part in the study without being forced. Signing means that you accepted to take part without being forced.

For me I think, for example even in the small Sacco’s we are part of, people are very quick to saying they have accepted to take part but again they are very quick to changing their mind due to any situation as you know our everyday life, they might get a challenge from else were and they start accusing the researchers saying “they even forced me to join the study” but if they completed that consent form and signed it very well, the researchers have proof and evidence on their side that that person agreed to take part and even signed the document, that is why we sign on those documents.

Signing that document means that I have agreed to every procedure that the researchers has told me about during the information session, and agreed to everything that will be done to me during the research, and that in all sudden situations they have agreed to be a part of it.

To sign, it shows that you have accepted the things they have asked you to do. The signature is an indication that you have accepted, you understand, or you have refused.

It’s important because that person who puts a signature on that document confirms that they have understood every information you have shared with them and agreed to participate in the study.

I feared because I don’t know where document with my name is going.

WHY A VOLUNTEER MAY REVIEW THE CONSENT DOCUMENTS?

There are various reasons that women may refer to the information document which implies that they take it seriously. Several reasons were shared including checking the procedures that were mentioned at the start of the study. Concern for breast milk draw and blood draws from a newborn were of concern that drew some women to check for what was written in the document. The implication here is that this is an important document.

When I realised that my baby was getting on and of cough and flu, I was forced to go back and review the consent form to check if they had included something on the side effects of the vaccine to the baby. When I reviewed it and didn’t find anything related to side effects, I then realised that I could be a normal flu. I thought my baby having on and off flu was an effect of the vaccine.

Since I was pricked several times [took off her blood sample several time], I also wondered if my baby would also be pricked several time like me. So I had to go back and review the consent form to check if she will also be pricked several times. When I looked through the form, they had put that they would draw some blood from the baby but it would not be often like us.

Personally, I didn’t go through everything in the consent form because I reached late but I informed them that I would go back home and read through the information again. One time I wanted to use the hand bag where I had kept the forms so when I checked in that bag and found those forms, I decided to read them.

Fac: You also reviewed the consent form. What motivated you to review it.

I reviewed that consent form several times because I wanted to better understand the kind of research I had participated in. I could review those consent forms with my husband. We could collect all the consent forms and reviewed them together.

I also went back and reviewed the papers because I wanted to know the duration of the study.

I reviewed the consent form because I was wondering why they were giving us money yet they were also taking off blood.

For me when they told me to extract breast milk the very first time in the study, I asked the doctor why they were taking my breast milk, the doctor told me that all that information was explained to me during the information session before I joined the study, I told him I understand that you taught me and I understood. He explained using a medical term the reason why they take off the breast milk on the first day to see whether…... I have forgotten the scientific word he used, but in brief that forced me to go back and look critically at the copy they gave me here, that’s how I understood that its true they had to collect breast milk on the first day I gave birth.

I used to read it every time after my study visits to find out what I will be doing on my next study visits when I go back to the hospital, that why I used to revisit it.

I read it several times when they had just given it to me, then I kept it some were safe, I had seen everything even collection breast milk on the first day, but what I didn’t see, was collecting blood from my baby, after two weeks they tell us to bring the baby and they take off a blood sample from their little hand, that really hurt me and I thought to myself ‘why do they take off all that blood from such a little baby’, I was puzzled; I went back and picked the paper and read it so critically to see whether it was in that paper , I found the information there and understood why the doctors did it.

For me the doctor told me after pushing you baby make sure they don’t cut off the umbilical cord before giving them blood from the cord, I asked myself ‘I thought this blood from the cord is bad, now why do they want it?’ I went back to the document they gave me and checked if it was there and it was clearly documented.

**Int:** What about others, didn’t you read the document again?

For me I used to come with it in my bag every time I had a visit, I used to take my time reading it while waiting to see the doctors, it helped me follow through very well, I could tell that this is what they are doing at this stage, just to prove that what they are doing is what they taught me in the beginning.

WITNESS

A witness in the consenting process is inclined to protect future occurrence of problems in case of any event. It is viewed as a legality kind of document, to protect research and the researcher. On a less note this person is to ensure they listen in to the information shared to the volunteer. However, some participants did not think it is necessary to have a witness, understanding overrides reading and writing by the volunteer.

The role of a witness is to provide proof that the participant consented to take part in the study without being coerced. The role of the witness is to help with signing for the participant who can’t write.

I think the witness is important in cases where that person who signed or put their finger print in agreement to participate in the study initially, later changes their mind about anything against the initial, they can ask the witness because they were around when that person agreed to participate in the study, and they can give evidence that that person was not forced to sign on that document.

Although the witness is said to be important there was a FGD participant who felt they are just like a witness in the bank and therefore not important. And understanding is not only tagged to reading and writing. During elections thumb prints are used and that is sufficient.

The role of the witness is to testify that a person understood what the study is about by putting his/her signature.

According to me, her point is weak because many times we go to the banks to sign documents with people who cannot write but there is an option of putting a thumb print. I think a witness can even reach a point and denies that she signed on your behalf. According to be, a witness is not important.

A person may not be able to read and write buy she understands. That means that her thumb print can allow her take part in the study. She can put a thumb print because it is the one we use during elections.

Some things need to be hidden. I may have my own secrets that don’t require me to be with a witness.

This witness must be able to understand the information not the same as the participant, meaning she should be able to explain it further to the participant, so you have to be clear when you identifying one.

The partial witnesses are important because participants understand better when they have someone in their shoes explaining to them.

The role of the witness is to provide evidence that the participant wasn’t forced to join the study. The witness would just testify that he/she was present when the participant was taking part in the study.

That witness will be present when you are reading the study information to the participant up to the time of signing. Therefore, in case of a problem, the witness will help to provide evidence.

I think the Importance of the witness is to prove that all the procedures were followed correctly; the witness provides evidence that the researcher explained to them and they understood before they accepted to take part in the study and they are also important to ensure that researcher does not take advantage of the participants.

Yes, it’s really important, that we have that person that process, because in case something happens, they would really be accountable, they should be able to understand the language used and able to read and write, so that at the end of the day they don’t put the blame on the medical personnel in case anything happens.

The role of a witness to people who can’t read and write, I don’t know.

She can explain to her the study information. In case of any problem, the witness can stand in and say that she was present.

The witness is the person who understands on your behalf. Because if he refuses, you don’t go ahead to it.

**Getting witnesses however is not always easy**

Like you have said, sometimes getting a suitable witness is not easy, sometimes we use hospital staff, remember they also have things to do yet going to pull her out of their work for one hour, just to seat and listen to you. I think what we can do for some of those cases is we can give them some time to go home and look through that information, and if they have someone at home who is literate, they can even ask them for help, then when they come back it would be a quicker process for them.

COMPENSATION OF PARTICIPANTS

But some women also have issues with this reimbursement, some feel like its little, others feel like why should they be paid yet they are volunteering, they believe that they are being bribed to be involved in the study, they think we are giving them that money for the blood we have collected from them.

Compensation for the participants first of all, reimbursing their transportation costs, because we call them from their places of work or their homes to come and carryout these study activities. Then another way we can compensate is through providing immediate medical care

We can offer them with some transport allowance as compensation. This can motivate them since they leave their work to come and participate in research. I want to appreciate you because you have been compensating people for their time.

**What should be the compensation?**

The respondents couldn’t define compensation. It differed from each individual but most suggestions were pointing to volunteers being given some money. There were some suggestions for things in kind such as a delivery kit, some food and any appropriate odder for the new born to support the care.

It’s tricky because the economy now is bad, so It is hard to come up with a general figure, but on the other hand having a general figure is good like thirty thousand, however the studies should also be flexible, and ask people where they have come from to come for the visit, the journey someone has travelled should be put into consideration

Ensuring that she gets the best midwife readily available. Also providing some requirements for the labour suit in case the mother is poor.

Int: What requirements do women use in the labour suit?

Like a cloth they use to wrap a baby after delivery. You can help her pay for such requirements. For my case, I know money more than anything else.

There is no best way to compensate for those participants even if you choose to give them one million shillings. You may not have all that money. You can seat as researchers and decide on what to give them.

You may offer a mama kit to that woman or offer her with other requirements required during delivery. You can also offer them with some T-shirts and a bag. Am grateful the MUJHHU team did that to us last time.

It’s very important for you to compensate the participants, because for example we have been seated here for some time like one hour, during this time I have spent with you I would do so many other things, so if my time is not compensated it would be unfair.

Apart from giving them cash, you can may be buy some items for the baby or some home necessities like you do some shopping for the family like food that is necessary for the woman after giving birth.

I think you have as a study thought about the best way which is giving them some money for transport when they come, this gives them the courage to continue taking part in the study, because sometimes they may not have enough, for example for me if my wife was not given transport to for study visits I would have stopped her already but the good thing she gets transport always. Transport motivates them a lot.

My opinion is quit wide; I think the researcher should first consider the distance between the study site and the participant’s home before they determine their transport reimbursement costs, they should also consider that fact that these prices keep on changing and then make an arrangement of increasing for the participants as well, they should also consider the time participants spend at the study site, especially for pregnant women because they are sensitive and can’t spend a long time without eating yet some studies don’t provide eats for these women when they turn up for study visits.

CONFIDENTIALITY what does it mean?

I think confidentiality means, keeping my secret about participating so that am not despised for people to say that am like a rat they use to test drugs, because that’s how people perceive it, yet they don’t know that it’s to benefit every pregnant woman.

It means that If you approach me for my views and I openly tell you, it means that this message is going to stay between the two of us.

It makes you keep safe because the information is only known to you alone since there are no names allocated. Information may be released but none will know that I am the one who said it.

The information shared by the participant must remain confidential in a way that they have to keep the information to only those people in the study, if I get information about a participant, am not supposed to tell it to a college in a funny way.

Confidentiality is actually keeping information, as private as possible, any personal information concerning the participants should be kept safe between a few study staff. And some of the information like personal identifiers should be kept under lock and key, and if we have a participant file, and you want to up load it, you have to conceal that information before you can up load it, like in redcap, you can upload the participants date of birth and it appears like XXX in the system, yet it is hidden data.

According to me, I don’t know how I have grown-up but; I don’t think confidentiality is necessary, I am a very straight person, I don’t think there is any information I want to hide, am so free even if my name is mentioned in public, especially if I know that I don’t have any crime, the only thing I mind about is protecting my family, so they can put my name anywhere, but they shouldn’t put other details like my Wife’s name and children, there I think it can help, me I don’t have any problem with that, for me they have my permission of putting my name on my views, or I can even come to speak as an advocate in case they want to encourage a particular group, I am proud of my opinions and I can stand out for them publically. Confidentiality is not so necessary at a certain extent.

Confidentiality helps to keep the dignity of the participant. This is because there are some people who answers the question arrogantly. So if you keep all her responses confidential, you maintain his dignity.

I agree that all the information shared by the participants should be kept confidential, what I mean is no one should be able to identify that these particular views came from the woman leader KF or attaching our names on views because, this means that we shall be identified, however you can just say that these views are from the leaders of Kawempe in general so that we don’t miss out on sharing our views with you, I don’t see anything wrong with that.

We are identified with numbers, they call numbers, they don’t call the name of a person. That helps us in case you fear other people to get to know about your participation. The identification numbers they give to us, there are the ones that helps us to remain confident, that the information we give, even if it goes public, it not easy to know that this and such a person is the one who gave out the information.

When they are providing study information to us, they tell us that statement that the information collected will be kept confidential were no one can identify me as one who provided it. That is why they don’t include my phone number and the place where I stay to the information I provide. The information is useful because it helps other people to know our experience in research.

I think they are not lying but I think some information may link, because I don’t know where they keep that information they take from us, I don’t know how you keep it, but for me I just trust the words you tell us, because we even give you our names.

I think it means keeping all the information within the hospital and not putting it on the internet; then ensuring that the information shared stays with in and is not share with other facilities like the joint medical stores.
